# Global trends and topics in CDK7 inhibitor research: a bibliometric analysis

**DOI:** 10.3389/fphar.2024.1426988

**Published:** 2024-09-25

**Authors:** Jiamin Liu, Ling He, Wenjing Jiang, Ping Xie

**Affiliations:** Department of Gynecology, Hospital of Chengdu University of Traditional Chinese Medicine, Chengdu, China

**Keywords:** VOSviewer, Citespace, bibliometric analysis, keyword analysis, trends

## Abstract

**Background:**

CDK7 has been demonstrated to play a crucial role in the initiation and progression of malignancy. Therefore, targeting CDK7, which regulates the transcription process, has emerged as a new promising approach for treating cancer. Research on CDK7 inhibitors has significantly increased over the past 2 decades, with almost 600 related papers in the Web of Science Core Collection database. To effectively identify future research hotspots and potential future directions, it is crucial to systematically review and visually present the research on this topic from a comprehensive viewpoint, ensuring scientific reliability.

**Methods:**

This study performed bibliometric analysis via CiteSpace and VOSviewer scientometrics analysis software to examine data on the publication of articles on CDK7 inhibitors over the past 2 decades; the data included country of publication, author names, institution names, scientific categories, cited journals, and keywords related to the field of CDK7 inhibitors.

**Results:**

This bibliometric analysis included 426 publications from 41 different nations, referencing a total of 15,892 sources. Research associated with CDK7 inhibitors has rapidly expanded since 2016, and the US and China are the two countries with the highest publication output among the countries and institutes that produce literature on CDK7 inhibitors. Furthermore, the US is the country that most frequently engages in international cooperation. The evolution of keywords identifying antitumor strategies related to CDK7-mediated cellular transcription processes has been the research focus in recent years.

**Conclusion:**

In this study, we identified research efforts and their evolving patterns and predicted advances in the CDK7 inhibitor field. The knowledge structure of CDK7 inhibitors encompasses pharmacological mechanisms, therapeutic targets, and cancer treatment strategies. The primary objectives of contemporary research are to discover the processes underlying cancer progression, identify specific signaling pathways, and develop effective clinical medicines.

## 1 Introduction

Cancer refers to many diseases characterized by the development of abnormal cells that divide uncontrollably and can infiltrate and ultimately perturb normal cell fate. The cell cycle is an exquisite cell-autonomous trait that serves as an internal control for regulating normal cell proliferation. When tumor cells undergo cell division, they adapt so that they can divide even under unfavorable conditions (Peissert et al., 2020; Li et al., 2022). Thus, cell cycle proteins and cyclin-dependent kinases (CDKs) have been investigated intensively in recent years for cancer research. This pioneering work demonstrated that CDK7, which is a critical member of the CDK family and a subunit of the transcription factor TFIIH, plays dual roles in cell cycle control (Attia et al., 2020; Chen et al., 2020; Guarducci et al., 2024) and transcription (Sun et al., 2020; Yuan et al., 2022). CDK7 functions in cell cycle regulation as a CDK-activating kinase (CAK) and is also involved in the control of transcription initiation and elongation via the phosphorylation of the C-terminal domain (CTD) of RNA polymerase II (Pol II). CDK7 is ubiquitously expressed, and interestingly, its protein level moderately increases in malignant tumor cells compared to control cells (Kim et al., 2020; Tang et al., 2021). Thus, previous studies have proven that CDK7 is an interesting cancer target and have provided valuable insights into cancer therapy.

In the G1 phase, CDK7 inhibitors can inactivate CDK2 and delay S phase progression (Fisher, 2005). Furthermore, in the S/G2 phase, CDK7 inhibitors block CDK1 activation and halt mitosis. In addition, CAK, a component of the transcription factor IIH (TFIIH) plays a role in regulating transcription (Martinez et al., 1997; Schachter et al., 2013). CDK7 inhibition could serve as an opportunity to press the pause button for oncogene transcription initiation and sustained transcription activation. Moreover, the activities of many transcription factors, such as p53 (Kalan et al., 2017), estrogen receptor (Chen et al., 2000), and androgen receptor (Russo et al., 2019), are also regulated by CDK7-mediated phosphorylation. Because CDK7 plays crucial roles in the malignant properties of cancer cells and regulates transcription-associated chromatin modifications, the field of CDK7 inhibitor research is booming. According to the characteristics of clinical trials for CDK7 inhibitors registered at ClinicalTrials.gov, several phase I/II trials of CDK7 inhibitors have been conducted in solid tumors. The recruited patients had breast cancer, colorectal cancer, lung cancer, prostate cancer, pancreatic cancer, small cell lung cancer, etc. The highly selective CDK7 inhibitors include CT7001, SY-5609, SY-1365, XL-102 and Q901, etc. SY-5609 is the only one approved by the U.S. Food and Drug Administration (FDA). In accordance with the latest European Society of Medical Oncology (ESMO), following the demonstration of robust antitumor activity in preclinical models of KRAS mutant patient-derived xenograft (PDX) models and ovarian cancer xenografts, a phase I clinical study to determine the preliminary clinical activity of SY-5609 was conducted. The clinical results revealed that 30% (11/37) of the response-evaluable patients had stable disease (SD) as the best response. Six SD patients had 8.7%–18.1% reductions in tumor volume, with a median of 198 d (range: 55–273) on treatment (Papadopoulos et al., 2020; Johannessen et al., 2021; Sharma et al., 2021).

Despite encouraging findings from preclinical studies of CDK7 inhibitors, further studies are needed to prove that patients may benefit from the clinical application of CDK7 inhibitors. Scientific research and preliminary clinical studies of CDK7 inhibitors are ongoing, and extensive bibliometric analysis and publication of a detailed overview on the basis of the current findings of CDK7 inhibitors remain to be performed. This study focused on the research hotspots and publication trends associated with CDK7 inhibition via the Web of Science (WoS). We used CiteSpace (Chen, 2006; 2014) and VOSviewer (Van Eck and Waltman, 2010; Arruda et al., 2022), both of which are Java-based software for metrological analysis and information visualization, for a comprehensive analysis of CDK7 inhibitor studies published from 2004 to 2023 and to visualize the current status and hot spots in this field. Therefore, our work is the first to characterize the global publication landscape of CDK7 inhibitors. We also aimed to simplify the landscape, map frontier research hotspots and explore recent trends in the evolution of CDK7 inhibitor research. This study provides a reference for research in the field of cancer therapy and is also helpful for promoting pharmacological and clinical medicine research on the basis of integrated analysis.

## 2 Materials and methods

### 2.1 Data source and literature search strategy

The bibliometric analysis data used in this study were downloaded from the WoS database (https://www.webofscience.com/). WoS is not only a comprehensive bibliographic database but also a live database with real-time data updates; its contents include journals, books, and science and technology proceedings and are constantly expanding (Pranckutė, 2021). We performed a static bibliometric analysis of publications related to CDK7 inhibitors in the Web of Science Core Collection (WoSCC). After conducting a thorough and integrative literature search on CDK7 inhibitors, we collected data on CDK7 inhibitor-related articles updated until 31 December 2023. We referred to the relevant reviews of CDK7 inhibitors (Sava et al., 2020; Li et al., 2022) and consulted experts in searching and evaluating scientific literature; all the authors agreed on the following systematic search strategy for the retrieved relevant articles: TS=(ICEC0942 OR BS-181 OR LY3405105 OR LDC4297 OR SY-1365 OR THZ1 OR THZ2 OR YKL-5-124 OR QS1189 OR SY-5609 OR CT7001 OR CDK7 inhibitor). To prevent potential bias in the data evaluation and updates due to data reliability, the literature search, extraction, processing and download steps were all performed by the same investigator on the same day. The literature type was limited to “article” and “review” from 2004 to 2023, and only articles with formal written English were acceptable. From the exported articles, we documented the authors’ names, study sources, titles, keywords, and cited references to avoid errors in retrieving articles at different times. All WoSCC records with cited references were extracted into tag-delimited plain text files.

### 2.2 Data analysis and graph acquisition

CiteSpace and VOSviewer were used to perform bibliometric analysis, and bibliometric network visualization of CDK7 inhibitors was conducted with the latest versions of R and RStudio with the installed bibliometrix package (version 4.1.4). We obtained information from PubMed for statistical purposes to better understand the research frontiers and perform hotspot analysis of CDK7 inhibitors in cancer treatment. ArcMap (version 10.8) was used to visualize the frequency of collaboration between countries. Microsoft Excel 2019 and GraphPad Prism 9.3.1 were used to construct the data tables and the graphs.

Additionally, the data were imported into VOSviewer (version 1.6.20) to generate network diagrams. The network was constructed utilizing bibliographic data, and coauthorship, co-occurrence, citation, and other forms of analysis were chosen on the basis of the content and nature of the analysis. Full counting was utilized for each count, and papers with many authors were not considered. The VOSviewer program was utilized to determine the number of publications and citations and the frequency of keywords. Visualization and construction of co-occurrence networks of crucial keywords from scientific literature were achieved with the help of the embedded clustering algorithm that was included in the software. This study’s primary focus was co-occurrence analyses, which constituted the bulk of this investigation. This tool was utilized to analyze collaboration between countries, institutions, and authors. CiteSpace allows the identification of highly cited references as well as keywords with large increases in the number of citations during a certain period.

## 3 Results

### 3.1 Overview of publication status

**FIGURE 1:**
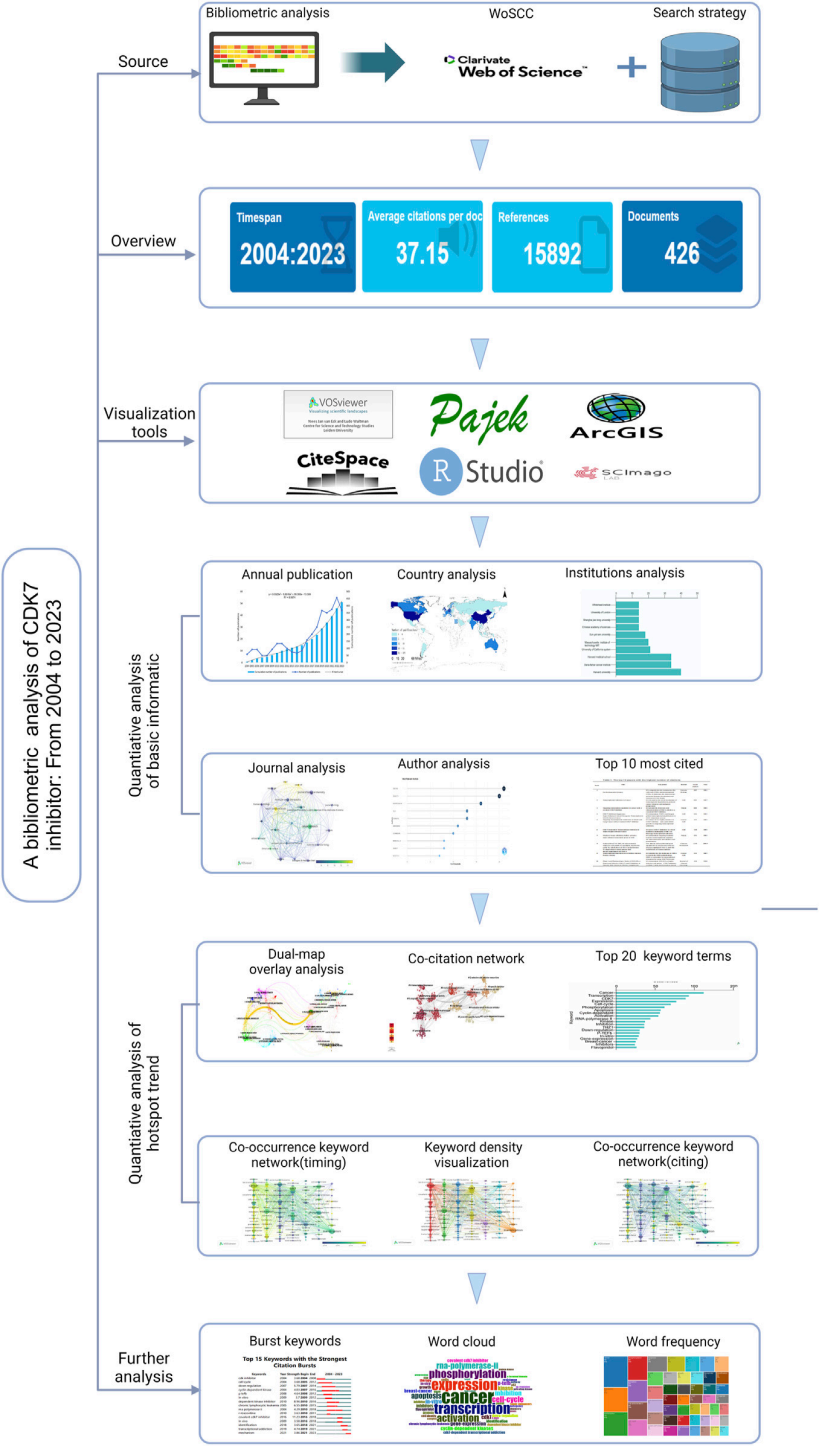
Schematic representation of the workflow used in this study.

Bibliometrics and citation analysis led to cybermetrics via scientometrics and informetrics. Web-based data help researchers understand their interactions and reasonably predict future research behavior. As shown in Figure 1, we performed a comprehensive bibliometric analysis of CDK7 inhibitors on the basis of a schematic representation of the workflow. A total of 579 records were included from the initial retrieval. We set criteria for the period (2004.01.01–2023.12.31, 535 studies left), literature type (Article or Review, 427 studies left), and language (English, 426 studies left). Finally, 426 studies were included in the review of the field of CDK7 inhibition (2004–2023). Key information is summarized in Table 1 from data retrieved from WoSCC.

**TABLE 1:** Main information about the data.

Description	Results
Documents	426
Article	368
Review	58
Time span	2004–2023
Sources (journals, books, etc.)	213
Annual growth rate (%)	9.89%
Document average age	6.88
References	15,892
Average citations per doc	37.15

### 3.2 Annual publication trends of CDK7 inhibitors

**FIGURE 2:**
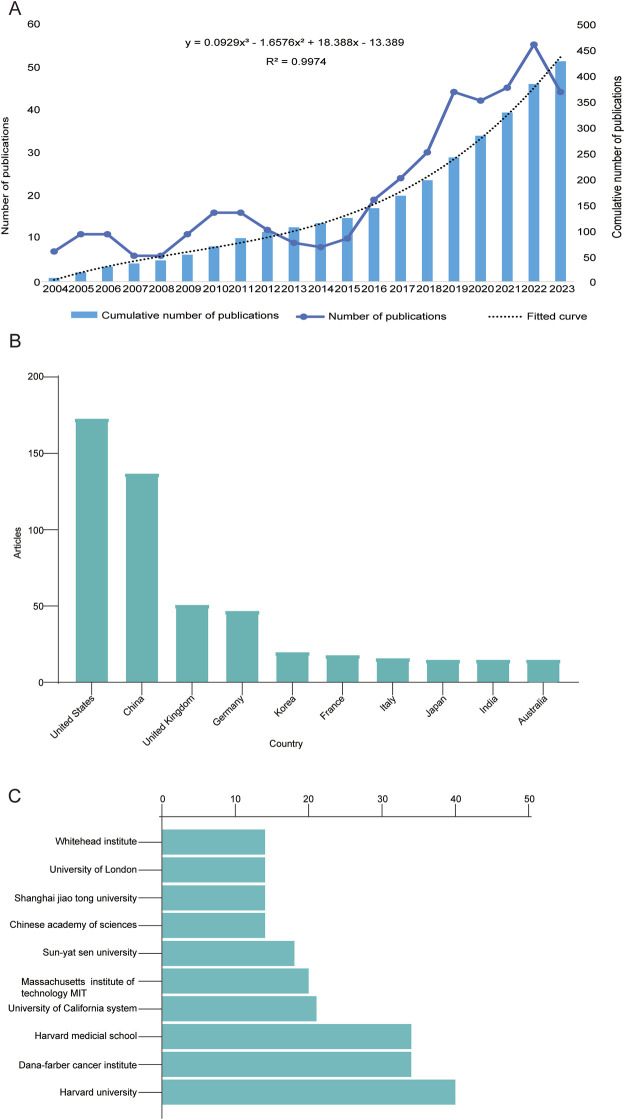
Quantitative analysis of publication status. **(A)** Annual volume of CDK7 inhibitor publications from 2004 to 2023. **(B)** Top 10 countries in the field of CDK7 inhibitors. **(C)** The top 10 institutions involved in CDK7 inhibitor research.

To explore the publication trends, annual publication numbers and cumulative numbers of publications related to CDK7 inhibitors, the publications were counted from 2004 to 2023. As shown in Figure 2A, the number of publications in the field of CDK7 inhibitors is increasing. With 7 publications in 2004 and 10 publications in 2015, little attention was given to CDK7 inhibitors in this period. As researchers realized the potential therapeutic value of CDK7 inhibitors, a rapid increase in related publications was observed in the following 8 years, with 55 publications in 2022. Moreover, through fitting regression analysis using the data from cumulative publications, the formulae for predicting the cumulative publications at different publication years were fitted. The R2 value of 0.9974 demonstrated the increasing trend and development of CDK7 inhibitor research. The number of publications on CDK7 inhibitors in the top 10 countries according to the WoSCC database (Figure 2B) is listed in order from highest to lowest. The U.S., China and the UK are the top three countries in this field, publishing more than all other countries combined. The details are listed in Supplementary Table S1. Furthermore, the top 10 institutions in terms of publication volume were also evaluated; similar to the distribution of publications by country, five institutions from the United States produced more than 20 publications on CDK7 inhibitors. Harvard University leads this field with 40 publications (Figure 2C).

### 3.3 Analysis of partnerships and cooperation among nations

**FIGURE 3:**
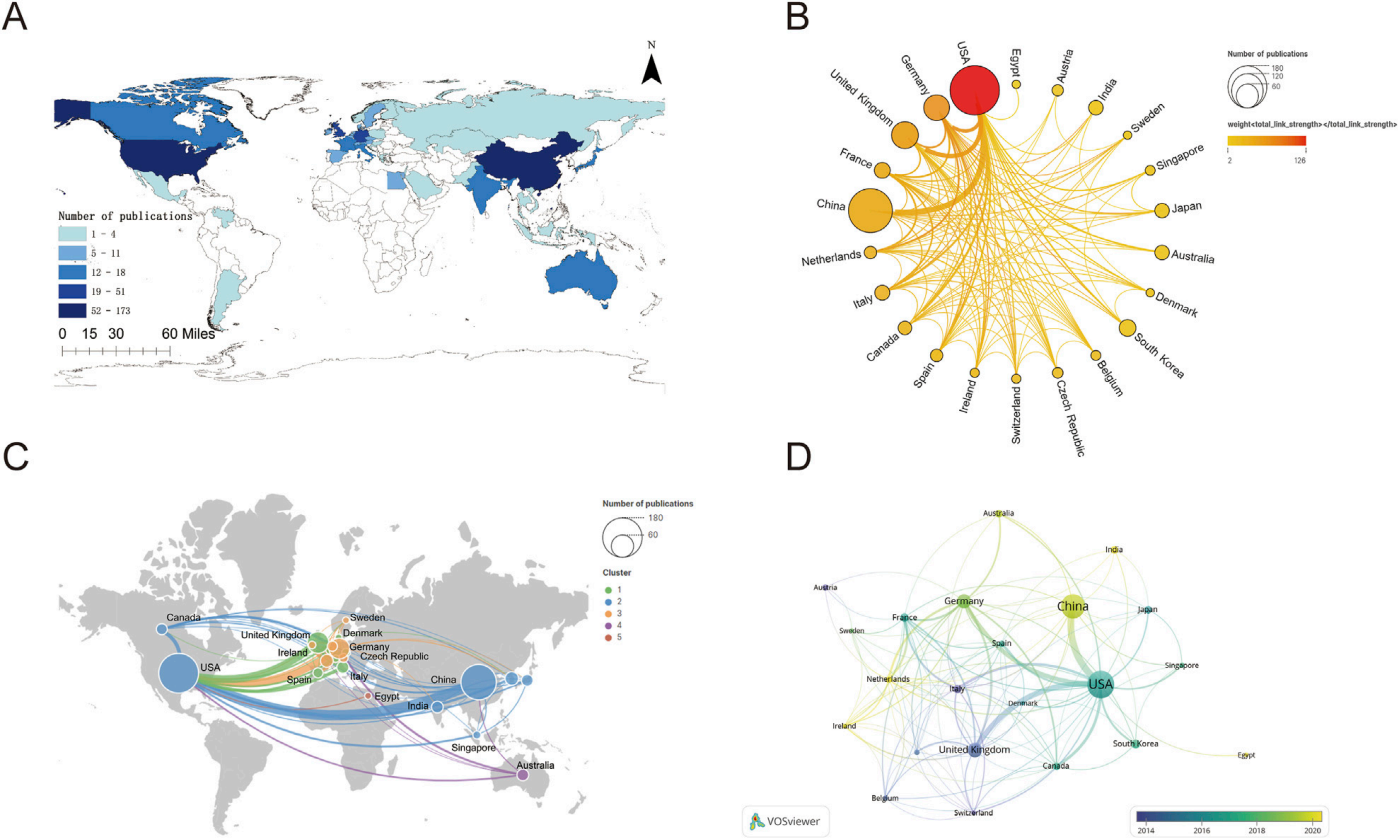
Assessment of partnerships and cooperation among various countries. **(A)** The filled map of 41 countries contributing to research on CDK7 inhibition. **(B)** Publication volume and cooperation intensity (collaboration network) of 22 countries in the field of CDK7 inhibition. **(C)** Citation frequency and number of collaborations among 22 countries that studied CDK7 inhibitors. **(D)** Cooperation trends in various countries from 2004 to 2023.

National publication counts were analyzed on a world map via ArcGIS, depicting the 41 countries contributing to research on CDK7 inhibitors (Figure 3A), which have attracted the attention of researchers worldwide. Figure 3A shows a schematic diagram of the national publications. The United States was the most productive, with 173 publications, followed by China (137 publications), the United Kingdom (51 publications), Germany (47 publications), and the Republic of Korea (20 publications). The remaining countries/regions have fewer than 20 publications. Furthermore, the total collaborative work between countries/regions was visualized by VOSviewer and Scimago Graphica via a circular plot (Figure 3B) and a world map (Figure 3C), respectively. In Figure 3B, countries are symbolized by a circle, with the magnitude of the circle corresponding to the quantity of its publications. Hence, the overall level of collaboration among nations has increased clockwise, starting from Egypt and ending with the United States. This indicates that, after the United States, China has the most publications worldwide. The degree of collaboration among nations is denoted by the color of the circle: pale yellow and red indicate the minimum and maximum levels of intensity, respectively. Only 22 countries have cooperated with other countries. The United States has been involved in numerous international collaborative studies. These results indicate that the United States plays a vital role in CDK7 inhibition research. Evidence from VOSviewer (Supplementary Figure S1) has repeatedly revealed this fact. Figure 3C shows there are many collaborative studies between the US and China (with a frequency of 26). In addition to China, the countries that have cooperated with the United States in descending order are the United Kingdom (with a frequency of 20), Germany (with a frequency of 15), Canada (with a frequency of 9) and Italy (with a frequency of 8). The number of international collaborations between countries was highest for the US and China and lowest for other Asian countries. Comprehensive details are presented in Supplementary Table S2. To further explore the distribution of the countries according to research time, national publications were visualized (Figure 3D). The circle’s color indicates the year of publication for the countries, with deep blue and light yellow representing the earliest and the latest years, respectively. Ten years ago, most studies published in this field were from the United States; however, the distribution of studies began to shift toward China and Ireland in the last 5 years, which may be related to recognizing the clinical value of CDK7 inhibitors in the academic community. The same trend is also evident in the author analysis (Supplementary Figure S2). These data also reflect the cutting-edge academic influence of the United States.

### 3.4 The evolution of research disciplines

**FIGURE 4:**
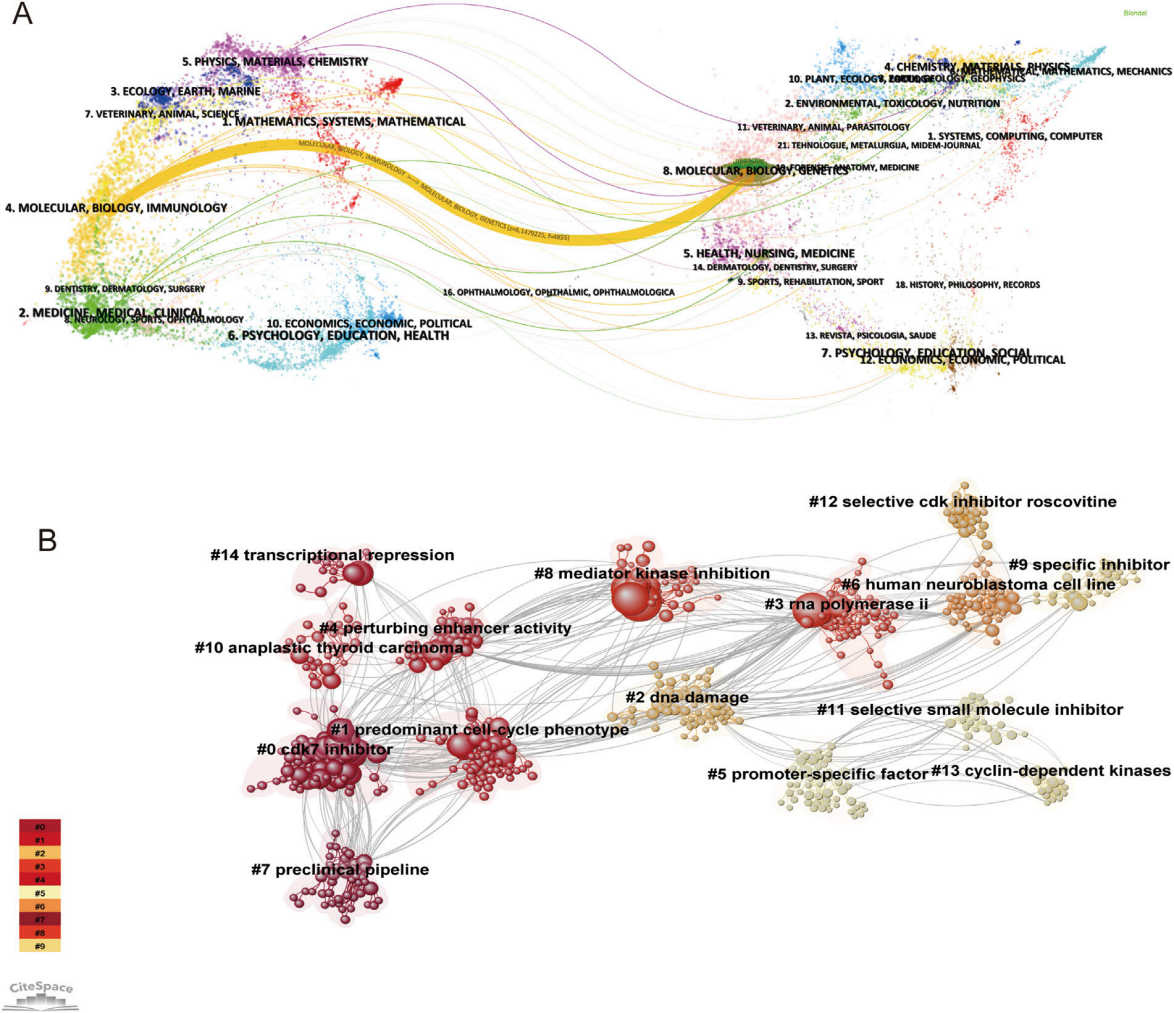
Analysis of the evolution of research disciplines and cocited references. **(A)** Dual-map overlay and corresponding disciplines. The citing journals are on the left, the cited journals are on the right, and the colored path represents the citation relationship. **(B)** Cluster analysis of cocited reference networks.

To visualize the interjournal citation relationship and the topic distribution of academic journals, we conducted a dual-map overlay analysis via CiteSpace. A basic graph of the citing journals is displayed on the left side of Figure 4A, and a basic graph of the cited journals is displayed on the right. Each dot on the map represents a journal. The curve is the citation line, which shows the full context of the citation. As shown in Figure 4A, the thickest line indicates that the core cited studies were articles on CDK7 inhibitors published in molecular/biological/immunology journals, and these studies were usually cited by molecular/biological/genetics journals. The trajectories of these lines reveal interdisciplinary crossover in the field.

### 3.5 Analysis of cocited references

The 426 retrieved publications contained a total of 15,982 cited references. References that are frequently cited in conjunction with other articles can be regarded as the foundation of research in a given field. It is possible to ascertain the background and knowledge base of CDK7 inhibitor research by examining cocitations among cited references. The software CiteSpace employs the log-likelihood ratio algorithm to determine the 15 largest clusters. Clusters were formed using the keywords extracted from references. These genes included “CDK7 inhibitor (Cluster #0, size = 202), “predominant cell cycle phenotype (Cluster #1, size = 187)”, “DNA damage (Cluster #2, size = 162)”, “RNA polymerase II (Cluster #3, size = 158)”, “perturbing enhancer activity (Cluster #4, size = 134)”, “promoter-specific factor (Cluster #5, size = 119)”, “human neuroblastoma cell line (Cluster #6, size = 95)”, “preclinical pipeline (Cluster #7, size = 87)”, “mediator kinase inhibition (Cluster #8, size = 73)”, “specific inhibitor (Cluster #9, size = 58)”, “anaplastic thyroid carcinoma (Cluster #10, size = 47)”, “selective small molecule inhibitor (Cluster #11, size = 34)”, “selective CDK inhibitor roscovitine (Cluster #12, size = 27, Silhouette = 0.886)”, “cyclin-dependent kinases (Cluster #13, size = 22)”, and “transcription repression (Cluster #14, size = 18)” (Figure 4B). Clusters #1, #2, #3, #4, #5 and #14 focused on the specific mechanism of CDK7 inhibitors in various solid tumors. Clusters #0, #8, #9, #11, #12 and #13 indicated the aliases of the CDK7 inhibitor. Clusters #6, #7, and #10 show the clinical value and therapeutic potential of CDK7 inhibitors.

### 3.6 Analysis of top-cited articles

The primary component of a bibliometric method is citation analysis, which serves as the origin of impact factors. Papers with a substantial number of citations are considered of great research importance. Highly cited articles constitute the cornerstone for determining the research direction of this subject. Table 2 presents the top 10 most highly referenced papers, with the number of citations presented in decreasing order. Most of the highly cited articles were published between 2014 and 2017. The top 10 highly cited articles that received the most citations had a total of 4,259 citations, ranging from 168 to 987. The analysis revealed that the review by Malumbres et al. (2014) comprehensively summarized the detailed role that CDKs play in controlling cell division and modulation of transcription and received the greatest number of citations (987, WoS score). It not only divides CDKs into cell cycle and transcriptional CDKs but also elaborates on the characteristic structural features, localization and function of the CDK family. Furthermore, Bradner et al. (Bradner et al., 2017) introduced the concept of transcriptional dysregulation in cancer cells and focused on the novel mechanism by which transcriptional dependency promotes cancer initiation and malignant progression. Moreover, 8 of the 10 studies investigated a promising therapeutic strategy involving the use of a CDK7 inhibitor as a potent antimalignant tumor agent and elucidated the underlying mechanisms of the drug-induced toxicity of CDK7 inhibitors in different solid tumors.

**TABLE 2:** Top 10 papers with the highest number of citations.

Rank	Title	Key points	Journal	Local citation	Year
1	Cyclin-dependent kinases (DOI: 10.1186/gb4184)	Comprehensively summarizes the cell-cycle CDKs and transcriptional CDKs. It elaborates the characteristic structural features, localization and function of CDK family	Genome Biology	987	2014
2	Transcriptional Addiction in Cancer (DOI: 10.1016/j.cell.2016.12.013)	Focused on the novel mechanism of transcriptional dependency promoting cancer initiation and malignant progression	Cell	683	2017
3	Targeting transcription regulation in cancer with a covalent CDK7 inhibitor (DOI:10.1038/nature13393)	Shows the discovery and characterization of THZ1, which is a covalent CDK7 inhibitor	Nature	604	2014
4	CDK7 Inhibition Suppresses Super-Enhancer-Linked Oncogenic Transcription in MYCN-Driven Cancer (DOI:10.1016/j.cell.2014.10.024)	Demonstrates THZ1 could target global transcriptional amplification in MYC-dependent cancers	Cell	436	2014
5	Targeting Transcriptional Addictions in Small Cell Lung Cancer with a Covalent CDK7 Inhibitor (DOI:10.1016/j.ccell.2014.10.019)	Finding shows that SCLC highly sensitive to CDK7 inhibitor and could inhibit growth by targeting transcriptional addiction	Cancer Cell	333	2014
6	CDK7-Dependent Transcriptional Addiction in Triple-Negative Breast Cancer (DOI:10.1016/j.cell.2015.08.063)	Proven CDK7 inhibition is a novel method challenging TNBC via blockage transcriptional addiction	Cell	310	2015
7	Mediator kinase inhibition further activates superenhancer-associated genes in AML (DOI:10.1038/nature14904)	Demonstrates targeting mediator kinases could negatively regulate SE-associated genes and inhibit AML proliferation	Nature	253	2015
8	Seliciclib (CYC202, R-roscovitine) induces cell death in multiple myeloma cells by inhibition of RNA polymerase II-dependent transcription and downregulation of Mcl-1 (DOI:10.1158/0008-5472.CAN-05-0233)	Finding shows that seliciclib-induced apoptosis in myeloma cells by downregulated Mcl-1 and the inhibition of transcription	Cancer Research	253	2005
9	Transcriptional Dependencies in Diffuse Intrinsic Pontine Glioma (DOI:10.1016/j.ccell.2017.03.011)	Identifies the SE landscape in DIPG to clarify the DIPG pathobiology. DIPG is vulnerable to transcriptional disruption by using CDK7 blockade	Cancer Cell	232	2017
10	Phase I and Pharmacologic Study of SNS-032, a Potent and Selective Cdk2, 7, and 9 Inhibitor, in Patients With Advanced Chronic Lymphocytic Leukemia and Multiple Myeloma (DOI:10.1200/JCO.2009.26.1347)	Demonstrates SNS-032, a highly selective and potent CDK7 inhibitor, in Phase I study shows modest clinical activity in heavily pretreated patients with CLL and MM.	Journal of Clinical Oncology	168	2010

AML, acute myeloid leukemia; CDK, Cyclin-dependent kinases; CLL, chronic lymphocytic leukemia; DIPG, diffuse intrinsic pontine glioma; MM, multiple myeloma; SCLC, small cell lung cancer; SE, superenhancer; TNBC, triple-negative breast cancer.

### 3.7 Frequency and clustering analysis of keywords

**FIGURE 5:**
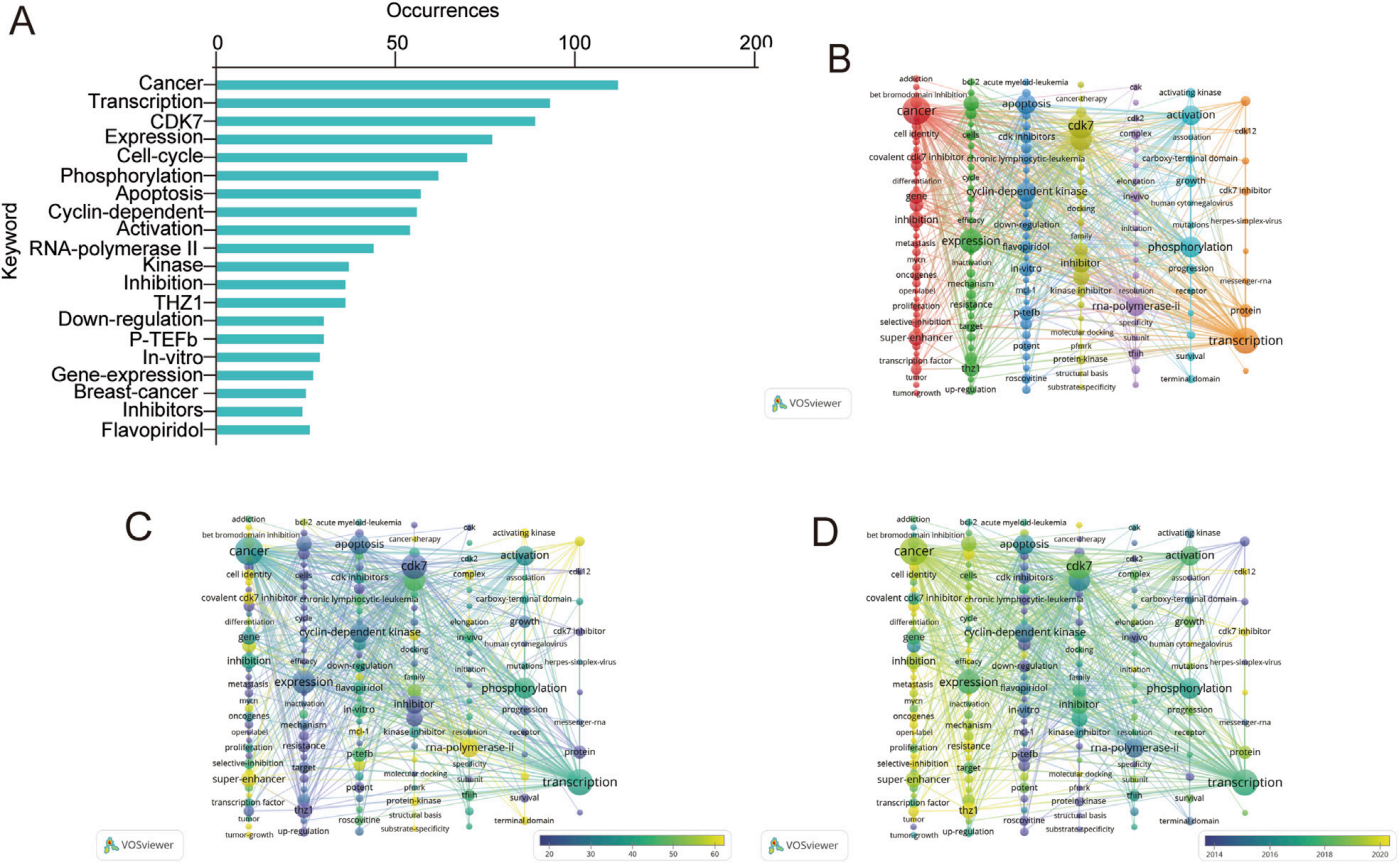
Network of related keywords in the study of CDK7 inhibitors (2004–2023). **(A)** The top 20 keywords in the field of CDK7 inhibitors. **(B)** Keyword cluster analysis. **(C)** Coexisting network of CDK7 inhibitors. **(D)** Temporal evolution of the co-occurrence keyword network.

The keywords used in each scientific publication represent its essence, and the co-occurrence of keywords precisely identifies the areas of research that are most active in this field. A total of 2,148 keywords were examined, 186 of which were found to meet the threshold of five occurrences. We merged these terms if they had meanings that were comparable to one another. The frequency-sorted top 20 terms are depicted in Figure 5A. Among these keywords, ‘cancer’ was the most commonly used, appearing 112 times. The next most common keywords were ‘transcription’ (N = 93) and ‘CDK7’ (N = 89). The term ‘apoptosis’ was mentioned 57 times, ranking seventh among the enriched terms. One of the most common cancer types, ‘breast cancer’ (N = 25), was among the top 20 keywords. The comprehensive information is shown in Supplementary Table S3.

The keywords were grouped into seven clusters. Closely related keywords were clustered together (Figure 5B). Group 1, represented in red, is related to the physiological role of CDK7 in different cancers. CDK7 has been demonstrated to play crucial roles in biochemical, physiological and molecular mechanisms; related keywords include “oncogene”, “cell identity”, and “metastatic”. Group 2, represented in green, was related to investigating the effects of CDK7 on phenotype, with keywords such as “resistance”, “cycle”, and “cells”. Some terms, such as “THZ1”, were also included in Group 2. Group 3, represented in blue, was related to the clinical application of CDK7 inhibitors, with keywords such as “flavopiridol”, “roscovitine” and “CDK inhibitors”. Group 4, shown in yellow, was related to cancer therapy combined with CDK7 inhibition, and the main keywords included “kinase”, “kinase inhibitor”, “protein kinase”, and “molecular docking”. Group 5, shown in purple, was related mainly to the role of CDK7 in transcription, associated with the transcription initiation factor TFIIH and the regulation of RNA Pol II-mediated transcriptional initiation and pausing; the keywords included “RNA polymerase II”, “initiation” and “elongation”. The light blue group, Group 6, was related mainly to the physiological role of CDK7. The orange cluster, Cluster 7, mainly included “CDK7 inhibitor”, “CDK12”, and “transcription”, which seemed to be related to the relationship between CDK7 and CDK12/13. For example, THZ1 is a covalent CDK7 inhibitor, and THZ1 can also irreversibly inhibit CDK12 and CDK13 (Greber et al., 2020). To determine the research hotspots and trends in CDK7 inhibitor research, the 186 keywords were colored according to the number of citations and the timeline. Figure 5C displays the citation-overlapping co-occurrence analysis network of keywords, and Figure 5D shows the time-overlapping co-occurrence analysis network of keywords. Every node in the graph corresponds to a certain keyword, the frequency of which is indicated by the node’s size. Additionally, the distance between the nodes indicates the strength of the relationship between the nodes. Figure 5C visualizes the overlapping citations of keywords. The least-cited keywords are displayed in blue, whereas the most-cited keywords are displayed in yellow. The most-cited keywords were “superenhancer”, “RNA polymerase II”, “covalent CDK7 inhibitor” and “cell identity”. The time-overlapping keywords are depicted in Figure 5D. Keywords that emerged early are shown in blue, whereas those that appeared more recently are shown in yellow. The initial investigations focused mostly on the topics of “transcription”, “RNA polymerase II”, “apoptosis” and “phosphorylation”. Contemporary research has focused on “THZ1”, “superenhancer”, “cell identity”, “CDK7 inhibitor”, and “oncogene”. Notably, through the changing patterns of keywords in recent years, the research focus in this field has evolved from the original emphasis on changes in physiological mechanisms to the clinical value of CDK7 inhibitors and potential therapeutic targets.

### 3.8 Further analysis of CDK7 inhibitors

**FIGURE 6:**
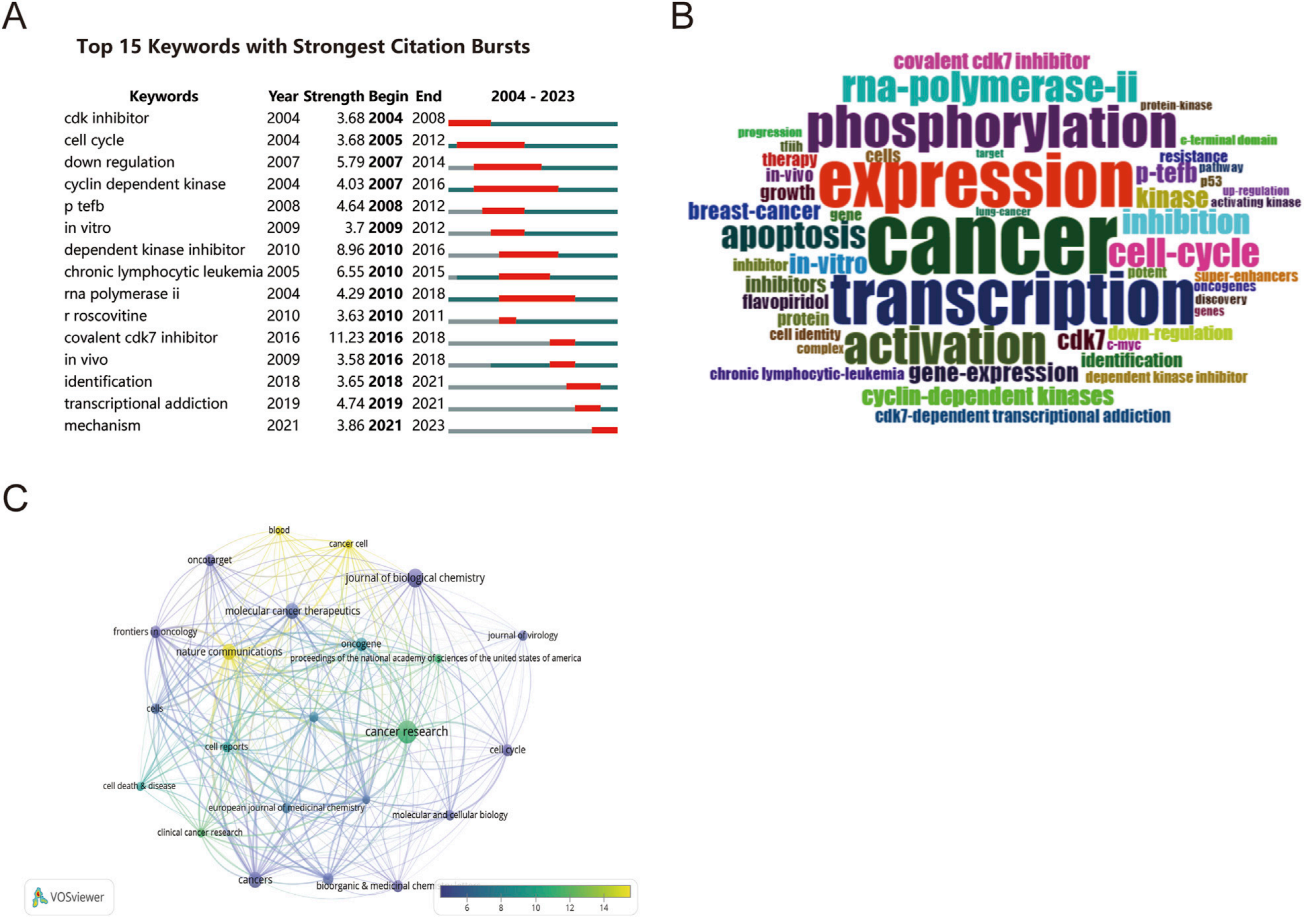
Further analysis of CDK7 inhibitors. **(A)** The 15 keywords with the strongest citation bursts. **(B)** WordCloud of keywords frequently used in CDK7 inhibitor publications. **(C)** Journal analysis of CDK7 inhibitors.

The keyword burst analysis is shown in Figure 6A. Through an integrated examination of the fifteen most frequently cited burst terms that have persisted for a minimum of 1 year, we found that the keyword “cyclin-dependent kinase” (2007–2016) garnered the most consistent attention. Keywords such as “identification” (2018–2021), “transcriptional addiction” (2019–2021), and “mechanism” (2021–2023) have recently been utilized. This may be related to the recent rapid increase in research between CDK7 and superenhancers, indicating that future research will focus on these keywords. To further establish a link between research trends and hot spots related to CDK7 inhibitors, a word cloud (Figure 6B) of keywords frequently used in CDK7 inhibitor publications was generated. These findings indicate that research on CD7 inhibitors has focused mainly on cancer, particularly on cell cycle phenotypes. Furthermore, the journals of published articles related to CDK7 inhibitors were analyzed via VOSviewer (Figure 6C).

## 4 Discussion

In recent years, compelling evidence has emerged that establishes the critical function of CDK7 inhibitors in impeding the initiation and progression of primary tumors (McDermott et al., 2020; Peng et al., 2021). In recent years, numerous works in the literature have been published in agreement with this concept (Zhang H. et al., 2020; Guarducci et al., 2024). Consequently, in this work, we utilized bibliometric methodologies to analyze the trajectory of research pertaining to CDK7 inhibitors from 2004 to 2023.

### 4.1 General information

A total of 426 articles regarding CDK7 inhibitors in the WoSCC were analyzed. Bibliometric R software, VOSviewer, and CiteSpace were used, and a comprehensive in-depth analysis of countries, institutions, journals, authors, and keywords was performed. The above tools were also used in this study to investigate the structure of knowledge, research hotspots, and emerging trends in the field. This study was performed to provide a foundation for the development of therapeutic strategies aimed at inhibiting CDK7. To advance medical research and clinical trials, improving our understanding of the role of CDK7 inhibitors in cancer treatment is crucial. This study is the first to perform a bibliometric analysis of CDK7 inhibitors in relation to the development and patterns of cancer treatment between 2004 and 2023. The increasing focus on the therapeutic potential and clinical benefit of CDK7 inhibitors in cancer patients is likely one of the primary factors contributing to the significant growth of research in this area over the past 10 years. Moreover, CDK7 inhibitors, which are capable of suppressing tumor growth, triggering cell death, and inhibiting the initiation of transcription in tumor cells, have become widely acknowledged as potent cancer treatments. This may explain the significant surge in the number of papers on this subject.

### 4.2 Analysis of leading countries and institutions

Growth in CDK7 inhibitor-related research can be divided into two stages. A phase of sluggish expansion prevailed prior to 2015, except 2010–2011, when sixteen studies were published. The maximum number of publications in subsequent years was twelve. Research associated with CDK7 inhibitors has entered a stage of rapid expansion, with the annual number of publications exceeding ten since 2016. By 2022, the yearly publication volume reached 55, which is an indication that research pertaining to CDK7 inhibitors has entered a phase characterized by swift advancements. Moreover, the U.S. and China have the highest publishing output among the countries and institutes that have produced literature on CDK7 inhibitors. Like the results for the distribution of publication numbers by country, five of the top 10 institutions in the United States produced more than twenty publications each. Only three universities in China were among the top 10, although China ranked second in terms of publishing counts. Furthermore, international cooperation with a focus on the United States is the most frequent form of collaboration. These findings validate the fact that the U.S. has made significant contributions and holds a leadership position in the field of CDK7 inhibitor research. This can be attributed to the nation’s favorable economic conditions and substantial investment in the medical field. This area will reap the benefits of broad international cooperation, which will ultimately lead to an improvement in the quality of research as a whole. Moreover, the number of publications originating from China and the United Kingdom is rapidly increasing, which may be a result of an increase in the size of the older population in these nations.

### 4.3 Hotspots and trends

Over the last 3 decades, there has been a significant shift in the principal fields of cutting-edge research, beginning with “molecular/biology/immunology” and progressing to “molecular/biology/genetics”, as demonstrated by a dual-map overlay study. The study of CDK7 inhibitors has undergone a significant transition as a result of these developments. Previously, the research focused on assessing molecular, biochemical, and immune-related symptoms and outcomes. Recently, most investigations have focused on genetic processes. These comprehensive transformations highlighted the overall direction and tendencies of development that had occurred. Furthermore, the background and knowledge base of CDK7 inhibitor research can be determined by studying the association between references. The various clusters include information regarding the unique mechanism of CDK7 inhibitors in a variety of solid tumors, as well as the effects of CDK7 inhibitors, their clinical utility, and their therapeutic prospects.

Keyword analysis is an extremely important tool for understanding the main topics of a publication, illuminating the dominating patterns within a specific subject, shedding light on the core topics of a publication, and assisting researchers in navigating prominent research fields. Within the scope of this investigation, our keyword co-occurrence analysis revealed that, during the last 3 decades, cancer, transcription, apoptosis, phosphorylation, and RNA polymerase II have been among the most common terms, showing that research has focused on cell identity, superenhancers, oncogenes, and the application of THZ1 in cancer therapy.

Previous research has demonstrated that THZ1, a very effective and promising inhibitor against tumors, can hinder cell invasion, induce apoptosis and migration in cancer cells, and modulate cellular signaling pathways (Huang et al., 2023; Jin et al., 2023; Tao et al., 2023). For example, THZ1 can kill bladder cancer cells by inhibiting the activity of superenhancer-associated oncogenes (Yang et al., 2021). Additionally, the inhibition of CDK7 could be a promising therapeutic approach to counteract immune evasion in cancer and improve the effectiveness of anti-PD-1 therapy (Wang et al., 2020; Li et al., 2024). The current study provides evidence that the CDK7 inhibitor THZ1 improves the effectiveness of anti-PD-1 therapy in nonsmall cell lung cancer by activating the p38α/MYC/PD-L1 signaling pathway (Wang et al., 2020). The importance of regulating CDK7 in cancer cell initiation, transcription, and resistance to therapy is now generally acknowledged.

Burst detection algorithms are efficient at identifying sudden surges in citations or keyword popularity within a brief timeframe, accurately identifying burgeoning study fields. The results of our research indicate that the first sudden increase in the use of the phrase ‘CDK inhibitor’ was observed in 2004. The research themes then broadened to include the cell cycle (Zhong et al., 2019), P-TEFb (Mbonye et al., 2018), and *in vitro* experiments (Clark et al., 2017). Owing to the increasing recognition of the role of CDK7 inhibitors in cancer treatment, research has focused more on understanding their actions and the mechanism by which they affect transcription addiction (Wang et al., 2015; Yuan et al., 2022). Since 2018, there has been a growing focus on identifying genes connected with superenhancers, as well as studying transcription addiction and its related mechanisms (Cao et al., 2019; Thandapani, 2019; Zhang J. et al., 2020). This trend has persisted until the present. These findings indicate that CDK7 inhibitors have garnered increasing interest in cancer research and are likely to remain a leading research topic. For example, Tarang et al. (Gaur et al., 2023) reported that the CDK7 inhibitor XL1024 reduces the phosphorylation of serine 2/5/7 at the carboxy-terminal domain of RNA polymerase II and via the CDK7/c-Myc/p53 signaling pathway, leading to lymphoma cell apoptosis. Numerous studies have also shown that CDK7 inhibitors can lead to cancer cell apoptosis (Zhang et al., 2019; Huang et al., 2023; Zhang et al., 2023). Consequently, further exploration is needed to investigate the potential mechanisms of CDK7 inhibitors. Moreover, we analyzed the evolution of keywords via a timeline map and found that identifying antitumor strategies related to CDK7-mediated cellular transcription processes has been the focus of research in recent years. Considering the results of burst detection discussed earlier, it is possible that clinical trials with CDK7 inhibitors and integrated strategies for treatment efficacy in many forms of cancer could be the direction of future research.

### 4.4 Limitations

This study focused on the current status of CDK7 inhibitors and provided guidance for subsequent research. This investigation is not without its limitations. Initially, only English-language articles recorded in the WoSCC database were included. The WoSCC database employs a stringent evaluation process to guarantee the inclusion of high-quality literature. This reference database is frequently used for bibliometric investigations. Because the WoSCC encompasses the overwhelming majority of high-quality studies (Chen et al., 2021; Liu, 2021), this has no effect on the general progress of the findings. Second, citation delays may have prevented recently published high-quality studies from receiving the attention they merited; therefore, these studies should be included in subsequent investigations. Third, the focus of our research was on material that has been published within the past 20 years. Therefore, scientists with an interest in the field should conduct additional analyses of the research newly published in 2024.

### 4.5 Implications for CDK7 inhibitor research

This study conducted a bibliometric analysis of studies related to CDK7 inhibitors from 2004 to 2023 via the WoSCC. The aim was to thoroughly characterize the current status of publications on CDK7 inhibitors, identify global research trends and hotspots, and offer a comprehensive and concise overview of the development of CDK7 inhibitors. First, generating a bibliometric map of studies offers references for researchers in this field. By summarizing and visualizing the current research directions and development trends of CDK7 inhibitors, clinicians and researchers can summarize past findings and ideas for future research. Second, in addition to providing researchers with a trustworthy foundation and direction for locating authoritative references and grasping research trends, mining neglected areas of research, identifying gaps in the literature, and providing ideas to resolve those gaps are also helpful. Third, we present a detailed account of the most recent discoveries in the field of CDK7 biology and the clinical application of CDK7 inhibitors for the treatment of cancer. Additionally, CDK7 inhibitors have shown strong anticancer activity in a variety of cancer subtypes, making them a potentially useful treatment method. As a result, researchers who are concentrating on treatment-resistant cancers that are now available may gain some insight from our findings. Fourth, this study will enhance academics’ understanding of nations that are receptive to collaboration and facilitate the establishment of new partnerships. Furthermore, it assists researchers in selecting suitable journals for publishing their studies, identifying relevant countries for collaboration, and accessing material related to the subject matter of their investigations.

## 5 Conclusion

In summary, this study provides a better understanding of the development, hotspots, trends, and frontiers of CDK7 inhibitor research and highlights areas that still require additional research as a valuable guide for researchers in this and related fields.

## Data Availability

The original contributions presented in the study are included in the article/Supplementary Material, further inquiries can be directed to the corresponding author.

## Supplementary material

SUPPLEMENTARY FIGURE S1
**(A)** Radar plot of the collaboration network in the field of CDK7 inhibition. **(B)** Cocollaborating country network.

SUPPLEMENTARY FIGURE S2 Author analysis in the field of CDK7 inhibition.
