# Supplementary figures and images for: Global trends and topics in CDK7 inhibitor research: a bibliometric analysis
Source: Front Pharmacol. 2024 Sep 25;15:1426988. doi: 10.3389/fphar.2024.1426988 (PMC11461233; doi:10.3389/fphar.2024.1426988)

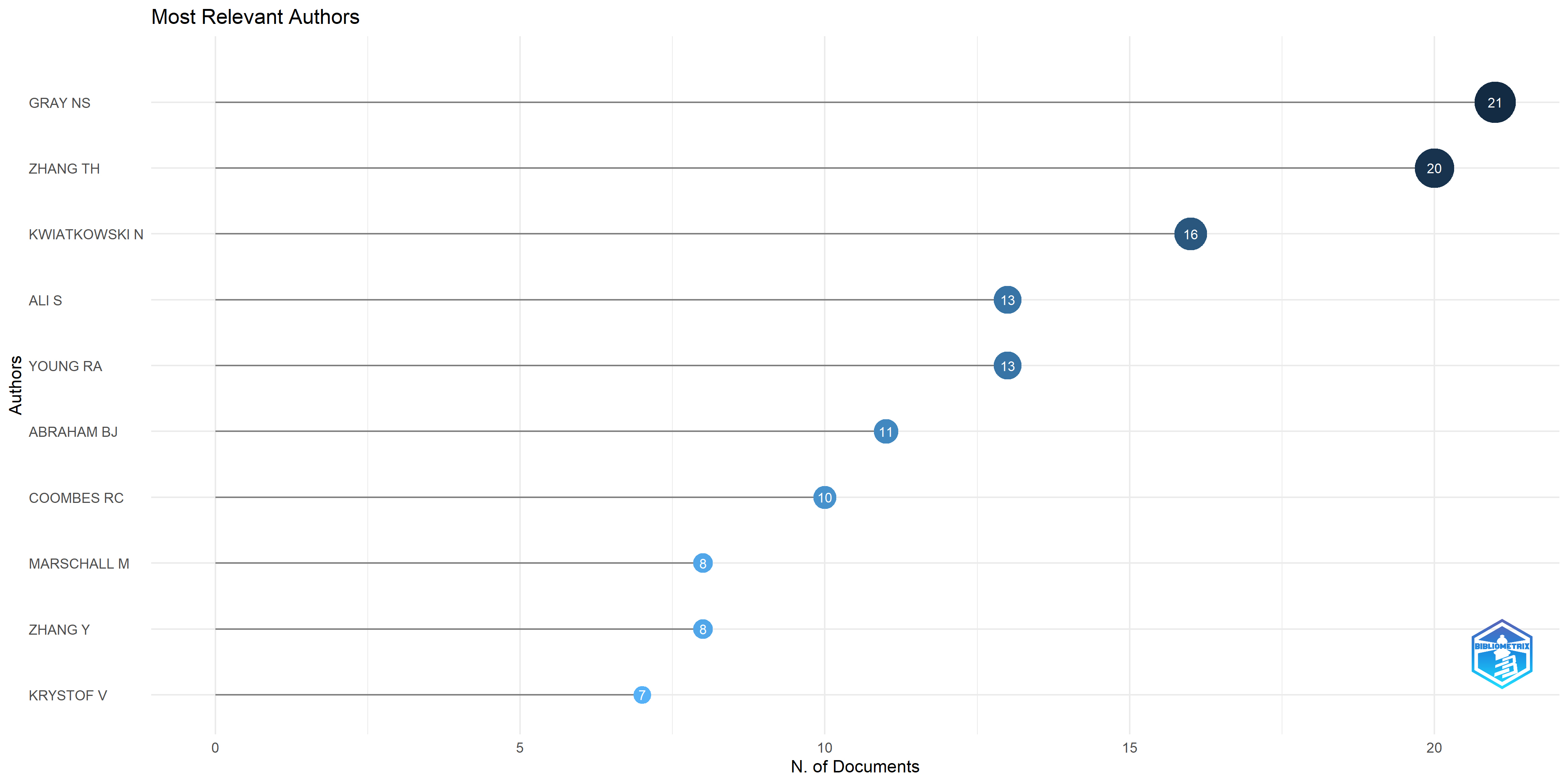

Supplement: Supplementary file 3 [file Image2.JPEG]

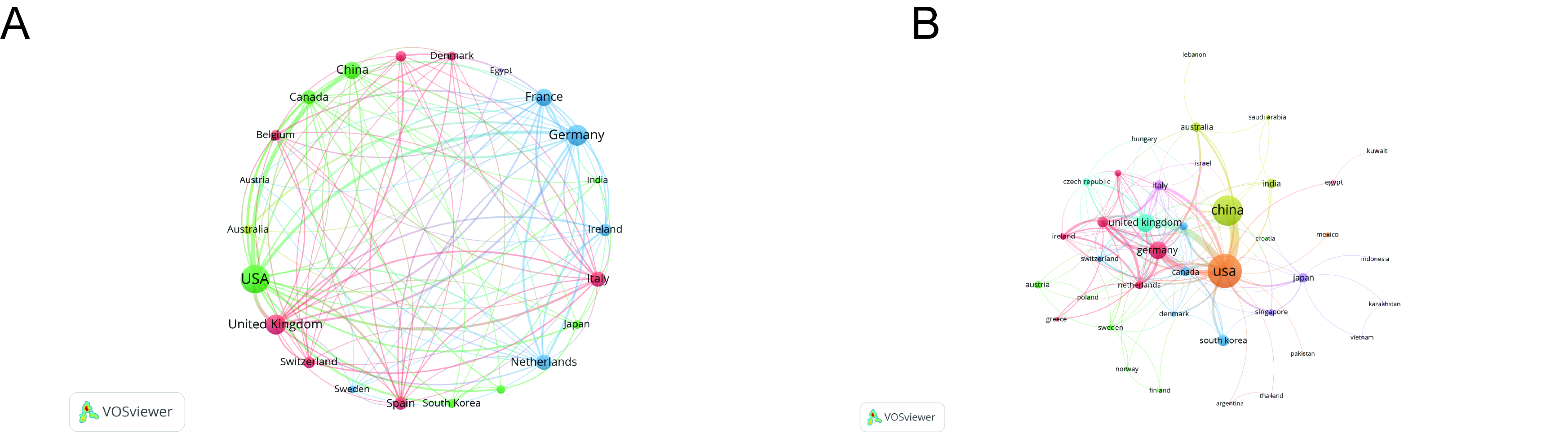

Supplement: Supplementary file 4 [file Image1.TIF]
